# Ovarian Collagen Regulates Granulosa Cell Proliferation Through Persistent Activation of the ERK1/2 Pathway in the Chinese Soft-Shelled Turtle (*Pelodiscus sinensis*)

**DOI:** 10.3390/ani16162499

**Published:** 2026-08-11

**Authors:** Xinqi Han, Yin Guo, Xiuyan Lou, Yanyan Zhang, Senhao Jiang, Chutian Ge, Ling Xiao

**Affiliations:** 1 Institute of Animal Sex and Development, Zhejiang Wanli University, Ningbo 315100, China; silvahan@163.com (X.H.); guoyin@zwu.edu.cn (Y.G.); 17280858237@139.com (X.L.); zyy1460485499@163.com (Y.Z.); 15888936587@139.com (S.J.); 2 College of Biological and Environmental Sciences, Zhejiang Wanli University, Ningbo 315100, China

**Keywords:** collagen, follicular development, granulosa cells, ERK1/2, *Pelodiscus sinensis*

## Abstract

The Chinese soft-shelled turtle (*Pelodiscus sinensis*) is an important aquaculture species in China, and proper follicular development is crucial for its reproduction. Collagen plays a critical role in ovarian development, yet its effects in this species remain unclear. Using primary granulosa cells and ovarian tissue, we found that collagen content varied dynamically throughout follicular development and peaked at the mid-vitellogenic stage. Collagen reduction resulted in granulosa cell detachment, inhibited proliferation, induced nuclear vacuolation, and arrested the cell cycle. Collagen reduction also caused a persistent increase in ERK1/2 phosphorylation over 24 h, which was associated with downregulation of *CCNE1*, *CDK2*, and *BCL2*; pharmacological inhibition of ERK1/2 phosphorylation partially restored the expression of these genes. These results indicate that collagen is involved in ovarian development, and its reduction may inhibit granulosa cell proliferation, at least in part, through persistent ERK1/2 activation. Our findings, obtained from an experimentally manipulated in vitro system, provide insights for in vitro culture of chelonian granulosa cells and ovarian tissues.

## Simple Summary

The Chinese soft-shelled turtle is an economically valuable aquaculture species in China, and its reproductive success is highly dependent on the normal development of ovarian follicles. In this study, we investigated the role of collagen, a structural protein that provides tissue integrity and mechanical support, within the ovary. We measured collagen levels at different stages of follicle development and observed dynamic changes, peaking during active vitellogenesis. To directly test the function of collagen, we reduced its levels in cultured ovarian tissues and granulosa cells. Following collagen reduction, granulosa cells lost normal adhesion, showed reduced proliferation, and exhibited structural damage. Concurrently, the ERK1/2 signaling pathway became abnormally active. Pharmacological inhibition of this pathway partially reversed the adverse effects induced by collagen reduction. Together, these findings suggest that collagen may play a role in regulating granulosa cell function in this species. Our results may inform the optimization of in vitro culture systems for reptilian ovarian tissues and contribute to better breeding strategies, ultimately supporting the aquaculture of this species.

## 1. Introduction

As reptilian species carry unique evolutionary traits and hold great aquaculture potential, their ovarian regulatory mechanisms remain largely underexplored. Among farmed reptiles, *Pelodiscus sinensis* (Chinese soft-shelled turtle) has been extensively cultivated nationwide in China and brings substantial economic gains to the aquaculture industry [1]. Stable follicle maturation acts as a decisive factor that determines breeding efficiency and production output. Follicular expansion is controlled by a complex network of local signaling molecules including hormones and cytokines, alongside mutual communication between different intrafollicular cell populations [2]. Normal oocyte growth and follicle maturation cannot proceed without effective crosstalk between follicular cells and surrounding stromal tissues, a biological process that fully relies on intact follicle structural architecture [3]. Within the follicular microenvironment, the ECM serves as a key determinant of follicle development.

Ovarian physiology depends critically on the structural and functional integrity of the ECM, which orchestrates key reproductive processes: follicle assembly, size expansion, steroid secretion, ovulatory rupture, and corpus luteum maturation [3,4]. Rapid volumetric growth of developing follicles requires synchronized structural reconstruction of granulosa cell basement membranes and adjacent ECM components. The ECM represents a dynamically adjustable biomolecular network that constantly reshapes its composition via protein deposition, structural modification, proteolytic breakdown, and molecular turnover [5]. Intact ECM architecture maintains regular ovarian tissue morphology and normal ovulatory capacity; in contrast, disordered ECM remodeling distorts ovarian mechanical properties and triggers tissue fibrosis, which eventually suppresses follicle growth and ovulation [6].

Collagen serves as the dominant structural constituent within ECM networks. In human ovarian tissues, type I collagen accounts for over 90% of all ECM protein contents [7]. This protein assembles into heterotrimeric complexes consisting of two α1 polypeptide chains encoded by *COL1A1* and one α2 chain transcribed from *COL1A2* [8]. As the primary structural scaffold of basement membranes, type IV collagen lies between epithelial granulosa layers and mesenchymal theca tissues [9]. Six distinct gene variants (*COL4A1* to *COL4A6*) encode six separate α chains (α1–α6 IV) that form type IV collagen molecules [10]. Granulosa cells continuously synthesize and remodel type IV collagen during follicle recruitment and GC proliferation, which creates suitable mechanical environments to support follicle enlargement [11]. Previous investigations have shown that collagen abundance undergoes dynamic fluctuations during follicle growth and corpus luteum formation [12,13].

Every mature ovarian follicle is composed of three key cellular layers: theca cells (TCs), granulosa cells (GCs), and the central oocyte. Granulosa layers wrap around oocytes and perform indispensable regulatory roles to support both follicle and gamete maturation [14,15]. Proliferation, differentiation, and apoptotic elimination of granulosa cells constitute core physiological events in the entire folliculogenesis process [16]. Within growing follicles, multi-directional communication between TCs and GCs, adjacent granulosa cells, as well as GCs and oocytes facilitates the exchange of signal molecules and metabolic substrates [17].

Numerous prior investigations have documented collagen’s essential regulatory roles in mammalian and avian ovarian systems, where it modulates granulosa cell proliferation, steroidogenesis, and follicle survival capacity [9]. Nevertheless, the existing literature rarely addresses collagen’s functional roles and downstream signaling cascades during follicular development in reptiles, despite their evolutionary significance and aquaculture value. The Chinese soft-shelled turtle provides an ideal experimental model to fill this research gap, given its economic significance in aquaculture and the well-characterized follicular developmental stages. In this research, we analyzed the expression of ovarian collagen and collagen-related genes across different follicle sizes. We also constructed an in vitro collagen reduction model using primary granulosa cells and ovarian tissues to explore how reduced collagen abundance affects granulosa cell proliferative ability and ovarian physiological function. Our findings offer novel mechanistic insights into collagen-regulated follicular maturation and provide a useful reference for future investigations in reptilian reproductive biology.

## 2. Materials and Methods

### 2.1. Animals

All animal handling operations strictly complied with the ethical standards issued by the Institutional Animal Care and Use Committee of Zhejiang Wanli University, China. Female *Pelodiscus sinensis* were purchased from a commercial aquaculture farm located in Ningbo (Mingfeng Aquafarm). Prior to tissue harvesting, turtles received anesthetic treatment and were sacrificed through cervical exsanguination.

### 2.2. Isolation and Culture of Follicular Granulosa Cells

The ovaries were collected from adult female *Pelodiscus sinensis* with an average body weight of 750 g to extract primary granulosa cells. All cellular experiments were performed with at least three independent biological replicates (*n* = 3), each derived from a separate batch of pooled cells. Fresh ovarian samples were harvested and rinsed three consecutive times using pre-cooled DPBS supplemented with a 1% penicillin-streptomycin mixture. Under sterile conditions, vitellogenic follicles measuring 5–8 mm in diameter were separated, and the theca shell was peeled off to isolate intact granulosa layers [18]. After eliminating residual yolk contents via DPBS rinsing, granulosa tissues underwent enzymatic digestion with 2 mg/mL type II collagenase (Solarbio, Beijing, China) in a 37 °C shaking water bath for 3 min; repeated pipetting was carried out during incubation to disperse cell clumps. Fetal bovine serum-containing DMEM was added to halt the digestion reaction. The cell mixture was filtered through a 70 μm cell strainer and centrifuged at 800 rpm for 8 min to gather cell pellets. After the supernatant was discarded, cell precipitates were resuspended in complete DMEM medium (Corning, Corning, NY, USA) consisting of 15% fetal bovine serum (Gibco, Waltham, MA, USA), 100 U/mL penicillin, 100 μg/mL streptomycin and 1% ITS (Sigma-Aldrich, St. Louis, MO, USA). Cell viability was assessed by the Trypan blue exclusion assay, with viability consistently maintained above 90%. Cells were seeded into 6-well culture plates (Nest, Wuxi, China) at a density of 5 × 10^5^ cells/mL and cultured at 30 °C with a 5% CO_2_ supply. A full medium change was performed 24 h post-seeding to wash away floating dead cells, and adherent cell growth status was monitored regularly. Culture medium was renewed every two days until cell confluence reached 90%, when various collagenase interventions were initiated. Before reagent administration, the old culture medium was aspirated, and cell monolayers were rinsed twice with DPBS, followed by cultivation in fresh DMEM supplemented with gradient concentrations of type II collagenase (Solarbio, China).

### 2.3. EdU and CCK-8 Assays

Following 24 h of collagenase treatment, 20 μM of EdU reagent (C10337; Thermo Fisher, Waltham, MA, USA) was supplemented into the culture medium, and cells were incubated for another 2 h to complete labeling. Cell staining was implemented according to the standard protocols attached to the Click-iT™ EdU Cell Proliferation Imaging Kit. Fluorescent signals were captured and imaged with a laser scanning confocal microscope manufactured (Nikon, Tokyo, Japan).

For CCK-8 proliferation detection, granulosa cells were seeded into 96-well plates in advance. Following 24 h of collagenase incubation, a 10 μL CCK-8 working solution (Solarbio, China) was dispensed into each culture well. The plates were kept at 30 °C for 2 h, and absorbance values at 450 nm were recorded to quantify relative cell proliferative activity.

### 2.4. RNA Extraction and RT-qPCR

Total cellular RNA was extracted from samples with TRIzol extraction kits supplied by Thermo Fisher Scientific (USA), strictly following the official operational manual of the product. Purified RNA templates were reverse-transcribed into complementary DNA using the K1691 reverse transcription kit from the same manufacturer. Quantitative real-time PCR reactions were set up with SYBR^®^ PrimeScript mix (Takara, Shiga, Japan) and detected on a Bio-Rad iCycler quantitative amplification platform. All amplification assays were repeated over three independent biological samples, while each cDNA template was analyzed in three technical parallel wells. Relative transcript abundance was quantified via the 2^−ΔΔCt^ algorithm, with GAPDH selected as the endogenous reference gene for internal normalization. Standard dilution curves were generated to calculate amplification efficiency for every primer pair, where valid efficiency values fell within the 90–110% range, and corresponding R^2^ coefficients exceeded 0.99. Target primers for *COL1A1*, *COL1A2*, *COL4A1*, *COL4A3*, *COL4A5*, *CCNE1*, *CCND2*, *CDK2*, and *BCL2*, alongside the reference primer *GAPDH*, were designed based on NCBI standard transcript sequences with Primer Premier 6.0 and Oligo 7 software. All oligonucleotide sequences were synthesized by Sangon Biotech (Shanghai, China) (Table 1).

### 2.5. Ovarian Tissue Culture and Chemical Treatment

Ovarian specimens were harvested from female *Pelodiscus sinensis* individuals averaging 300 g body weight, which carried follicles at early vitellogenic developmental phases. For each experiment, tissues from three independent turtles were used as biological replicates (*n* = 3). Under sterile working environments, ovarian tissues were cut into tissue blocks with a volume of roughly 5 mm^3^. After three rounds of DPBS rinsing, the tissue fragments were distributed into 24-well culture plates (Nest, China), with 2–3 tissue blocks placed inside every single well. Complete DMEM base medium containing 100 U/mL penicillin and 100 μg/mL streptomycin was added to each well, together with gradient doses of type II collagenase (Solarbio, China) or two ERK1/2 selective inhibitors (SCH772984, Selleck Chemicals, Houston, TX, USA; ERK1/2 inhibitor I, MedChemExpress, Monmouth Junction, NJ, USA). The culture plates were incubated in a cell incubator maintained at 30 °C and 5% CO_2_, in a constant humidified atmosphere.

### 2.6. Paraffin Sectioning and Masson’s Trichrome Staining

Collected ovarian tissues underwent 24 h fixation at 4 °C using 4% paraformaldehyde (PFA) solution. Post-fixation samples were rinsed and dehydrated with 50% ethanol before long-term preservation in 70% ethanol at 4 °C, reserved for subsequent paraffin embedding. For section preparation, fixed tissues went through gradient ethanol dehydration, xylene transparency and paraffin embedding procedures sequentially. Embedded tissue blocks were sliced into continuous 8 μm-thin sections. Masson’s trichrome staining kits (Solarbio, China) were utilized for collagen labeling according to the manufacturer’s standard protocols. Prepared stained slices were observed and imaged under a Nikon optical light microscope (Japan).

### 2.7. Immunofluorescence Staining

Paraffin-embedded ovarian tissue slices were dewaxed in xylene, rehydrated via a graded ethanol series of decreasing concentrations, and thoroughly washed with double-distilled water. For antigen retrieval, slides were submerged in 0.01 mol/L sodium citrate buffer and heated at 96 °C for 20 min. Next, tissue sections underwent 1 h blocking at room temperature with a sealing mixture consisting of 10% normal donkey serum, 3% BSA and 0.3% Triton X-100 diluted in 0.01 M PBS. Blocked samples were incubated overnight at 4 °C with goat anti-FOXL2 primary antibody (dilution ratio 1:200, catalog AB5096; Abcam, Cambridge, UK). After three 10 min PBST washing cycles, slices were incubated with Alexa Fluor 488-conjugated donkey anti-goat IgG secondary antibody (1:300, A21207; Invitrogen, Carlsbad, CA, USA) to visualize target antigens. Cell nuclei were counterstained with 286 nmol/L DAPI (Sigma, USA). Following final PBS rinsing, slices were sealed with coverslips and imaged with a Nikon laser scanning confocal microscope (Japan).

### 2.8. Western Blot Analysis

Ovarian tissues and granulosa cells were lysed in RIPA buffer (Solarbio, China) supplemented with mixed protease and phosphatase inhibitor cocktails. Cell lysates were centrifuged to eliminate insoluble precipitates. Total protein concentration of each supernatant was quantified via the BCA protein quantification kit (PC0020; Solarbio, China). Equal amounts of separated protein samples were loaded onto 7.5% or 10% SDS-PAGE gels for electrophoresis, then transferred onto PVDF membranes (Millipore, Burlington, MA, USA). Membranes were blocked with 5% BSA solution prior to overnight incubation at 4 °C with primary antibodies against the following targets: COL1A1 (1:1000, CQA8183; Cohesion, London, UK), COL4A1 (1:1000, CY1657; Abways, Shanghai, China), COL4A3 (1:1000, CPA5943; Cohesion, UK), COL4A5 (1:1000, CPA3177; Cohesion, UK), total ERK1/2 (1:2000, CY5487; Abways, China), phosphorylated ERK1/2 (1:2000, CY5277; Abways, China), total mTOR (1:2000, CY5306; Abways, China), phosphorylated mTOR (1:2000, CY6571; Abways, China). After primary antibody incubation, membranes were incubated with HRP-conjugated goat anti-rabbit secondary antibody (1:10,000, SA00001-2; Proteintech, Rosemont, IL, USA) at room temperature. Protein bands were visualized using an ECL chemiluminescence kit (Yamei, Shanghai, China). After signal exposure, membranes were stripped with commercial stripping buffer (Yamei, China) and re-probed with mouse anti-β-actin (1:10,000, 66009-1-Ig) or mouse anti-GAPDH (1:50,000, 60004-1-Ig; Proteintech, USA) as internal reference proteins under identical incubation conditions. ImageJ 1.54g software was utilized to calculate the gray values of target bands, and relative protein expression was normalized to β-actin or GAPDH signals.

### 2.9. Statistical Analyses

Each experiment was repeated at least three times to verify reproducibility. All quantitative data are presented as mean ± SD derived from at least three biological replicates. Statistical comparisons were implemented by one-way or two-way ANOVA, accompanied by Tukey’s post hoc multiple comparison test using GraphPad Prism 10 software. Differences with *p* values less than 0.05 were defined as statistically significant.

## 3. Results

### 3.1. Expression of Collagen in Ovarian Follicles

**Figure 1:**
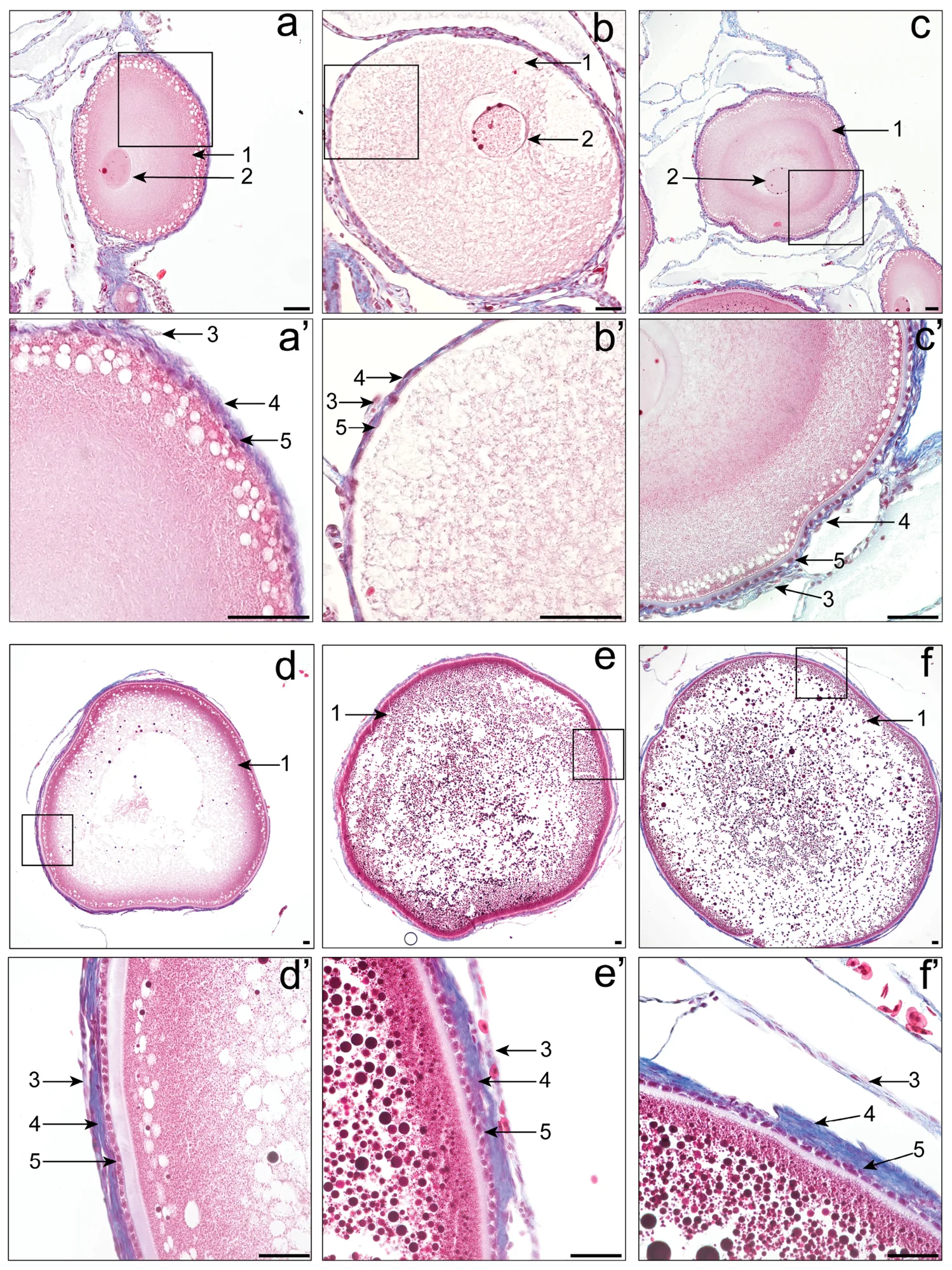
Collagen distribution (blue signal) in small follicles (<3 mm in diameter) visualized via Masson’s trichrome staining. (**a**) 0.33 mm, (**b**) 0.4 mm, (**c**) 1 mm, (**d**) 1.5 mm, (**e**) 2 mm, (**f**) 3 mm. (**a’**–**f’**) Enlarged views of the corresponding panels (**a**–**f**). Follicles shown in panels (**a**,**b**) represent early primary follicles enclosed by a single layer of flattened granulosa cells, whereas follicles in panels (**c**–**f**) are primary follicles surrounded by monolayer cuboidal granulosa cells. 1, oocyte; 2, nucleus; 3, theca cells; 4, collagen fibers; 5, granulosa cells. Scale bar = 50 μm.

To characterize collagen spatial distribution during early follicular growth in *Pelodiscus sinensis*, ovarian follicle slices from follicles below 3 mm in diameter were subjected to Masson’s trichrome staining for histological observation (Figure 1). This staining technique renders collagen fibers bright blue for clear visualization. Quantitative observation revealed a positive correlation between collagen accumulation levels and follicle size in follicles below 3 mm in diameter. For primordial follicles measuring merely 0.33 mm across, follicle walls remained thin, with faint, scattered blue collagen signals restricted to the outermost thecal layer; this phenomenon implied immature connective tissue frameworks within follicle walls at this early developmental phase. As follicles expanded to 0.4–1.5 mm in diameter, granulosa cells transformed from flat squamous epithelia into cuboidal morphology, a hallmark of follicle activation, accompanied by a steady rise in deposited collagen. When follicles grew to around 3 mm, their walls underwent obvious thickening and contained dense, intensely stained collagen meshwork, which constituted sturdy structural scaffolds to sustain subsequent follicle expansion and preserve tissue architecture. Collectively, these histological observations suggest that collagen accumulates stepwise throughout early folliculogenesis, and such dynamic matrix deposition is tightly linked to follicle enlargement, maturation and stable morphological maintenance.

### 3.2. Expression Patterns of Collagen-Related Genes in Follicles of Different Sizes

**Figure 2:**
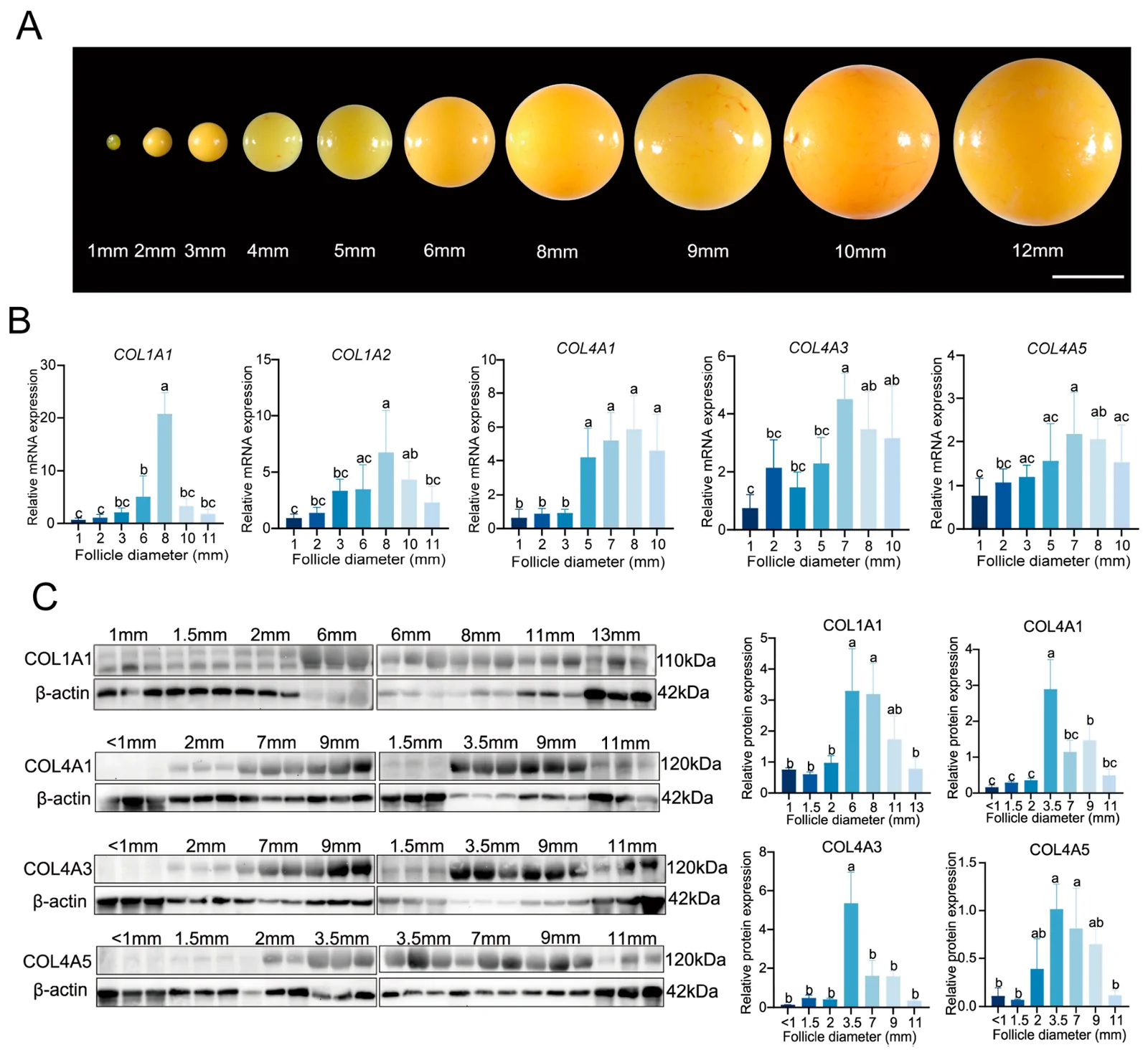
Expression of collagen-related genes during follicular development. (**A**): Ovarian follicles of different sizes in the Chinese soft-shelled turtle. Scale bar = 5 mm. (**B**,**C**): Relative expression of collagen-related genes in the follicles of the Chinese soft-shelled turtle. RT-qPCR analysis (**B**), *n* ≥ 3 biological replicates. Western blot analysis (**C**), *n* = 3 biological replicates. Statistical analysis: one-way ANOVA followed by Tukey’s multiple comparison test. Different lowercase letters indicate significant differences between groups (*p* < 0.05), while groups sharing the same letter are not significantly different.

To track dynamic fluctuations in collagen abundance across folliculogenesis in *Pelodiscuss sinensis*, ovarian follicles of varying diameters were harvested for combined mRNA and protein-level detection of collagen-associated genes. Figure 2A displays the morphological shift in follicles along developmental gradients: follicles expand from tiny translucent structures (1 mm diameter) to large yolk-laden yellow follicles reaching 12 mm in size. Prior to biomolecule extraction, yolk substances were fully cleared from isolated follicles, followed by collection of the thecal and granulosa cell layers. All tissue fragments were rinsed with DPBS prior to total RNA and total protein extraction. Quantitative RT-qPCR measurements showed a gradual upward trend in mRNA levels of *COL1A1*, *COL1A2*, *COL4A1*, *COL4A3* and *COL4A5* within follicles under 6 mm, with maximal mRNA abundance observed in 6–8 mm follicles (Figure 2B). Subsequent Western blot quantification further revealed that the protein levels of these collagen subtypes exhibited nearly identical expression trends to their corresponding transcripts and peaked in 6–9 mm follicles (Figure 2C). Taken together, these mRNA and protein profiling data suggest that ECM components undergo dynamic changes throughout follicle maturation of Chinese soft-shelled turtles.

### 3.3. Collagen Reduction Inhibits Proliferation of Granulosa Cells

**Figure 3:**
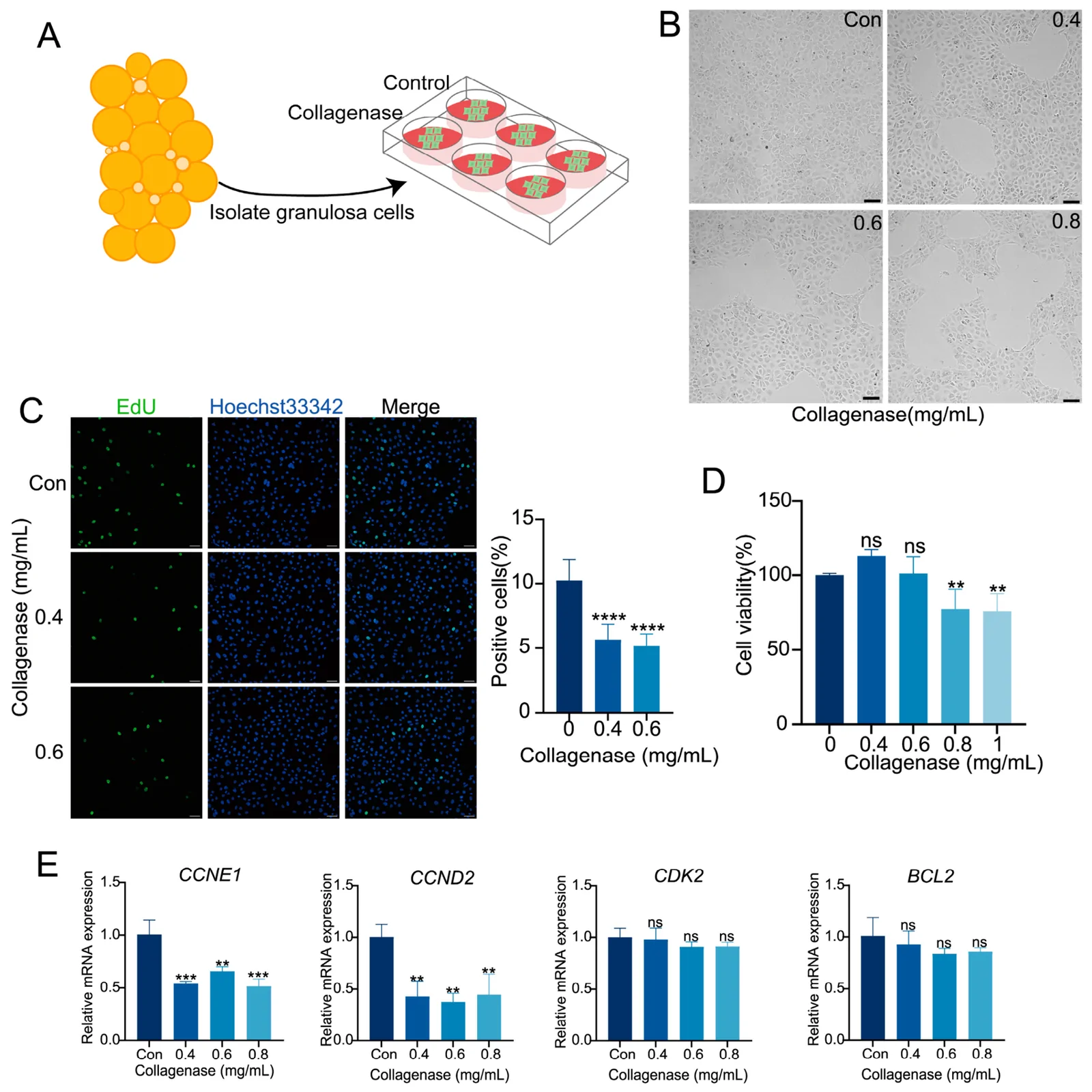
Collagen reduction suppresses granulosa cell proliferation. (**A**): Schematic illustration of the granulosa cell culture system. (**B**): Micrographs of granulosa cells incubated with 0, 0.4, 0.6 and 0.8 mg/mL collagenase for 24 h. Scale bar = 100 μm. (**C**): EdU proliferation detection in granulosa cells subjected to 24 h collagenase treatment. EdU-positive cells (green) denote proliferative cells; cell nuclei were counterstained using Hoechst 33342 (blue). Scale bar = 50 μm. (**D**): CCK-8 viability measurement of granulosa cells after 24 h of collagenase exposure. (**E**): Relative mRNA expression of cell cycle-related genes in the granulosa cells after treatment with collagenase for 24 h. Statistical analysis: one-way ANOVA followed by Tukey’s multiple comparison test; *n* ≥ 3 biological replicates. Statistical significance: ** *p* < 0.01, *** *p* < 0.001, **** *p* < 0.0001; ns, not significant.

Primary granulosa cells isolated from *Pelodiscus sinensis* were exposed to serially diluted type II collagenase to construct an in vitro collagen reduction model. This approach was adopted to investigate the regulatory association between collagen matrix and granulosa cell proliferation. Following 24 h treatment with 0.4, 0.6 and 0.8 mg/mL type II collagenase, granulosa cell detachment increased markedly in a dose-dependent manner (Figure 3B). Two approaches, EdU staining and the CCK-8 colorimetric assay, were applied to evaluate the influences of collagen reduction on cell proliferative activity. EdU staining showed that the proportion of EdU-positive proliferative cells decreased significantly under treatment with 0.4 mg/mL and 0.6 mg/mL collagenase (*p* < 0.0001, Figure 3C). Consistently, CCK-8 absorbance values indicated obvious reductions in cell viability in the 0.8 mg/mL and 1 mg/mL collagenase groups compared with untreated controls (*p* < 0.01, Figure 3D).

RT-qPCR analysis further revealed reduced mRNA levels of *CCNE1* and *CCND2* upon collagenase treatment (*p* < 0.01). In contrast, the mRNA abundances of *CDK2* and *BCL2* exhibited no significant differences among all experimental groups (Figure 3E). Collectively, these phenotypic and mRNA data suggest that collagen reduction induces granulosa cell detachment, attenuates proliferative capacity, and lowers the expression of core cyclin genes, thereby arresting normal cell cycle progression.

### 3.4. Collagen Reduction Is Associated with Cell Cycle Arrest in Ovarian Tissue

**Figure 4:**
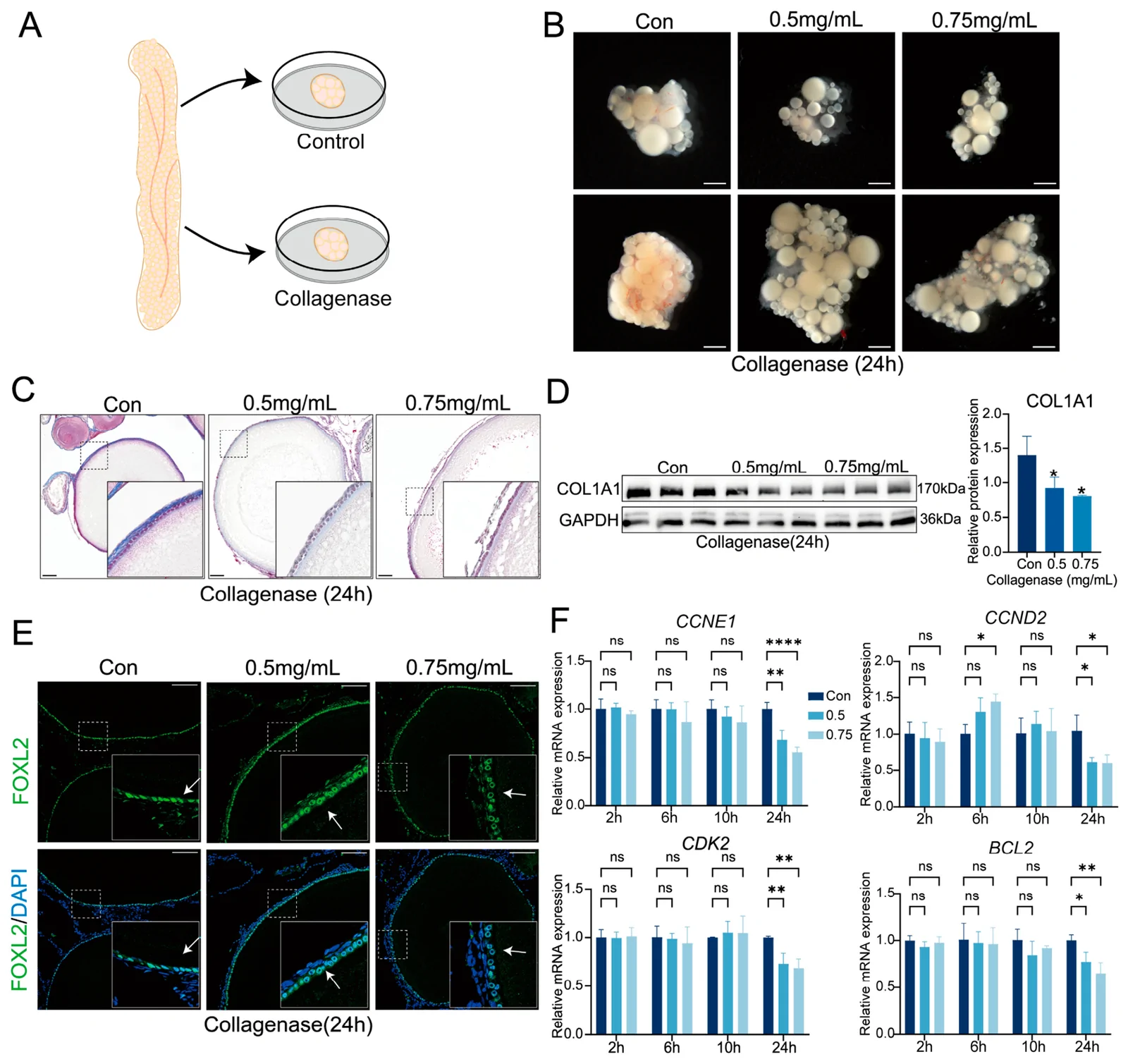
Collagen reduction within ovarian tissues correlates with cell cycle arrest. Ovarian tissues were cultured and treated with collagenase at concentrations of 0, 0.5 and 0.75 mg/mL for 24 h. (**A**): Schematic illustration of the ovarian tissues culture system. (**B**): Morphological characteristics of ovarian tissues following treatment. Upper and lower panels show ovarian tissues of different sizes (upper: small fragments; lower: large fragments), illustrating that tissue size modulates collagenase penetration and the degree of collagen reduction. Scale bar = 1 mm. (**C**): Masson’s trichrome staining of ovarian tissues after 24 h collagenase treatment. Blue signal indicates collagen fibers. Scale bar = 100 μm. (**D**): Relative protein abundance of COL1A1 in ovarian tissues. Statistical analysis: one-way ANOVA followed by Tukey’s multiple comparison test. (**E**): Immunofluorescent staining of treated ovarian tissues. FOXL2 (green) marks granulosa cells, and DAPI (blue) stains cell nuclei. Arrows indicate representative granulosa cells. Scale bar = 100 μm. (**F**): Relative mRNA expression of cell cycle-associated genes in treated ovarian tissues. Statistical analysis: two-way ANOVA followed by Tukey’s multiple comparison test; *n* = 3 biological replicates. Statistical significance: * *p* < 0.05, ** *p* < 0.01, **** *p* < 0.0001; ns, not significant.

To further characterize collagen function in ovarian follicles at the tissue level, ex vivo ovarian tissues were treated with collagenase to establish a collagen reduction model. Histological observation revealed disrupted tissue structure after collagenase incubation; looser intercellular organization was detected in treated samples, reflecting impaired endogenous collagen networks (Figure 4B). Masson’s trichrome staining offered supporting evidence, showing a notable loss of collagen-positive blue signals surrounding follicles in collagenase-exposed groups compared with untreated controls (Figure 4C). In line with these morphological results, Western blot assays showed significantly lowered COL1A1 protein abundance in ovarian tissues treated with 0.5 mg/mL and 0.75 mg/mL collagenase (Figure 4D). Combined morphological and protein data indicate the successful generation of an ex vivo ovarian tissue model featuring collagen reduction. Immunofluorescence imaging detected prominent nuclear vacuolation in FOXL2-positive granulosa cells isolated from collagenase-treated tissues (Figure 4E). Furthermore, RT-qPCR analysis revealed significant downregulation of cell cycle regulators (*CDK2*, *CCNE1*, *CCND2*) as well as the anti-apoptotic gene *BCL2* following 24 h collagenase treatment (Figure 4F). Collectively, these data suggest that collagen reduction induces morphological defects and cell cycle arrest in granulosa cells within ovarian tissues.

### 3.5. Collagen Reduction Inhibits Granulosa Cell Proliferation via the ERK1/2 Pathway

**Figure 5:**
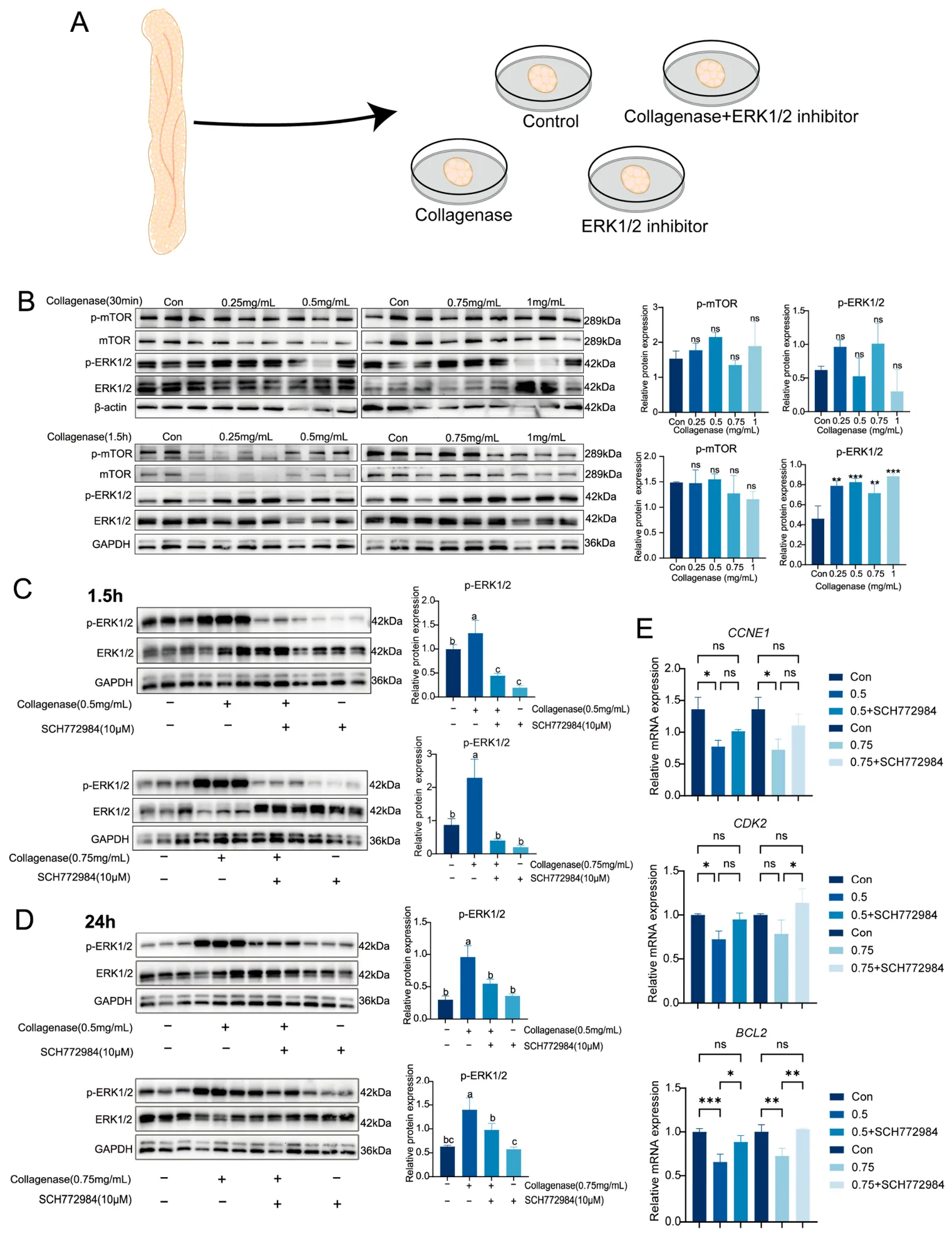
Blocked granulosa proliferation upon collagen reduction relies on activated ERK1/2 signaling pathway. (**A**): Schematic illustration of the ovarian tissue culture system. (**B**): Quantified phosphorylation abundances of mTOR and ERK1/2 in ovarian tissues after 30 min and 1.5 h collagenase incubation. (**C**,**D**): Changes in phosphorylated ERK1/2 abundance in ovarian tissues co-cultured with collagenase plus SCH772984 at 1.5 h and 24 h post-treatment. (**E**): Relative mRNA expression of *CCNE1*, *CDK2* and *BCL2* in ovarian tissues receiving combined collagenase and SCH772984 stimulation for 24 h. Statistical analysis: One-way ANOVA followed by Tukey’s multiple comparison test; *n* = 3 biological replicates. Statistical significance: Different lowercase letters indicate significant differences between groups (*p* < 0.05); * *p* < 0.05, ** *p* < 0.01, *** *p* < 0.001; ns, not significant.

To uncover the molecular cascade linking collagen reduction to suppressed granulosa cell growth, we assessed phosphorylation levels of mTOR and ERK1/2 signaling molecules after collagenase treatment of ovarian tissues. Time-series immunoblotting assays showed that 1.5 h collagenase incubation triggered robust upregulation of phosphorylated ERK1/2 relative to untreated groups, while total and phosphorylated mTOR levels exhibited no obvious alterations (Figure 5B). Such data imply the ERK1/2 cascade, rather than the mTOR axis, functions as a key mediator transmitting the anti-proliferative effects triggered by collagen reduction.

To investigate ERK1/2’s functional participation in this regulatory cascade, two selective ERK1/2 antagonists (SCH772984 and ERK1/2 inhibitor I) were applied for rescue assays. Western blot detection at the 1.5 h time point showed that co-administration of either inhibitor efficiently blocked collagenase-evoked ERK1/2 hyperphosphorylation (Figure 5C and Figure S1A). We then prolonged culture duration to 24 h to clarify how long ERK1/2 activation could last and verify the long-term inhibitory effect of the two reagents. The results showed that ERK1/2 phosphorylation stayed markedly higher at 24 h post-collagenase treatment, indicating this activation is persistent instead of transient (Figure 5D). Parallel RT-qPCR analysis also showed that ERK1/2 blockade could partially reverse the collagenase-mediated downregulation of *CCNE1*, *CDK2* and *BCL2* mRNA levels (Figure 5E and Figure S1B). Collectively, the experimental evidence suggests that persistent ERK1/2 hyperactivation over a 24 h window contributes to the proliferation suppression triggered by collagen reduction in *Pelodiscus sinensis*.

## 4. Discussion

Extracellular matrix (ECM) components exert multifaceted regulatory impacts throughout folliculogenesis, governing follicle growth, cellular survival, steroid synthesis, basement membrane assembly and oocyte maturation [12,19]. As the primary structural constituent within ovarian ECM networks, collagen carries dual physiological functions: maintaining tissue structural integrity and modulating diverse cellular behaviors [20]. The present work reveals dynamic shifts in collagen abundance across turtle follicle maturation and suggests that collagen modulates granulosa cell proliferation via the ERK1/2 signaling pathway.

Distinct collagen fiber morphology and thickness exist across different animal taxa. Previous mammalian research covering human, bovine and murine models demonstrated that follicle activation accompanies lowered collagen deposition around follicular compartments [21]. Studies on avian ovaries have recorded rising collagen concentrations as follicles expand in size [2]. Our histological data in *Pelodiscus sinensis* illustrated gradual collagen accumulation within follicles below 3 mm diameter. Sales and colleagues reported that type IV collagen predominantly localizes to basement membranes of the freshwater fish *Brycon opalinus*. Their work revealed high collagen abundance in resting and early-mature follicles, accompanied by reduced levels during late vitellogenesis [22]. Multiple prior investigations have also reported elevated matrix metalloproteinase (MMP) expression prior to ovulation, which degrades follicular collagen and reduces its overall content [23,24,25]. Our mRNA and protein detection data showed that transcripts encoding *COL1A1*, *COL1A2*, *COL4A1*, *COL4A3* and *COL4A5* increased in early-stage follicles, peaked at the 6–8 mm mid-vitellogenic phase, and declined afterward. Based on the established oogenic staging system of soft-shelled turtles, ovarian maturation can be divided into pre-vitellogenic and vitellogenic phases [26]. The observed mid-vitellogenic collagen peak may result from increased structural requirements driven by rapid yolk accumulation, which generates substantial mechanical stress on follicle walls. Combined with existing published evidence, these observations suggest that collagens are involved throughout follicle development and may exert more prominent regulatory functions during mid-vitellogenic stages in chelonians.

Granulosa cells serve as indispensable regulators of ovarian follicle development. Cytokines and functional proteins secreted by granulosa cells coordinate follicular expansion while supplying nutrients to developing oocytes [27]. Collagen shapes ovarian cell morphology to modulate metabolic activity, cell adhesion and migratory capacity [9,28]. In porcine granulosa cell culture, cells attach far more efficiently to substrates coated with type I or IV collagen than bare plastic culture surfaces [29]. Consistent with these published observations, our collagen reduction treatment dramatically weakened granulosa cell attachment capacity. This finding suggests that collagen may play a key role in sustaining stable cell–matrix crosstalk essential for normal granulosa physiological performance.

Collagen acts as a key modulator of granulosa proliferative activity. For instance, silencing the *COL1A1* gene in chicken preovulatory granulosa cells significantly suppressed cell division and triggered cell cycle arrest [30]. Granulosa cells cultured without collagen coatings readily undergo apoptotic death, whereas collagen-supported culture systems sustain long-term cellular viability [31,32,33]. Such evidence suggests that ECM collagen delivers vital pro-survival and proliferative signals lost once cells detach from matrix scaffolds. Disrupted granulosa cell–matrix interactions and reduced cell adhesion are linked to multiple ovarian disorders including polycystic ovary syndrome and premature ovarian failure (POF), which cause impaired reproductive capacity [34]. Our EdU and CCK-8 data showed that collagen reduction suppresses granulosa proliferation and induces nuclear vacuolation. The prior literature linked nuclear vacuolation to damaged nuclear envelopes; this morphological trait often precedes cellular senescence or apoptotic progression [35]. In addition, ovarian tissues subjected to collagenase digestion exhibited loosened tissue architecture, accompanied by significant downregulation of mRNA levels of the cell cycle mediators *CDK2*, *CCNE1*, *CCND2* and the anti-apoptotic gene *BCL2*. These outcomes support the hypothesis that lowered collagen abundance correlates with inhibited granulosa cell proliferation. Still, further experiments relying on TUNEL or Annexin V staining are needed to clarify whether reduced *BCL2* expression ultimately drives granulosa cell apoptosis under collagen reduction conditions.

Our experimental data illustrated rapid ERK1/2 hyperphosphorylation following collagen reduction in ovarian tissues, with this activated state persisting for 24 h. Co-incubation with the ERK1/2 inhibitor SCH772984 successfully blocked collagenase-triggered ERK1/2 activation. This treatment also partially restored the suppressed mRNA expression of *CDK2*, *CCNE1* and *BCL2*. These results suggest that the ERK1/2 signaling pathway may mediate proliferative suppression induced by collagen reduction. Nevertheless, ERK1/2 inhibitor treatment failed to fully restore gene expression to control levels. This observation implies that additional uncharacterized signaling axes also contribute to granulosa cell growth regulation alongside the ERK1/2 pathway.

Most existing studies reported that mild ERK1/2 activation facilitates granulosa cell proliferation [36,37,38], yet this growth-promoting effect only occurs under weak pathway stimulation [39]. By contrast, persistent, robust ERK phosphorylation has been reported to initiate cell cycle arrest and senescence across various cell types, frequently via upregulating cell cycle suppressors such as p53, p21^Cip1^ and p27^Kip1^ [40,41,42,43,44]. Similar regulatory patterns exist in *Xenopus* oocytes, where persistent ERK activation targets Cdc25A for degradation and halts cell cycle progression [39]. Collectively, published evidence highlights that the duration of ERK signal transduction, rather than signal intensity alone, dictates cellular fate [40,45]. In our turtle ovarian model, collagen reduction induced persistent ERK1/2 phosphorylation over 24 h. This change coincided with lowered cell cycle gene expression and impaired granulosa proliferation. It is therefore reasonable to infer that prolonged ERK1/2 hyperactivation contributes to the anti-proliferative phenotype observed under collagen reduction.

Several limitations should be acknowledged for the current research. First, collagenase treatment represents an experimentally induced model rather than a naturally occurring biological process. Second, all experimental phenotypes were obtained using in vitro manipulated models, which cannot fully recapitulate native ovarian physiological environments. Isolation of granulosa cells from intact follicular microenvironments and enzymatic collagen digestion inevitably generate artificial culture conditions that differ from the in vivo ovarian microenvironment. In addition to specific collagen breakdown, type II collagenase treatment may trigger unforeseen off-target biological effects. Third, although our findings provide mechanistic insights into collagen-regulated granulosa cell function, the proposed regulatory model requires further validation through complementary approaches, including gene knockdown, gene editing, and in vivo assays. Accordingly, all conclusions drawn from this work must be interpreted within the boundaries of the current in vitro experimental setup. Collagen exerts context-dependent regulation of granulosa cell behavior, and our in vitro findings may not fully recapitulate endogenous ovarian regulatory pathways.

## 5. Conclusions

**Figure 6:**
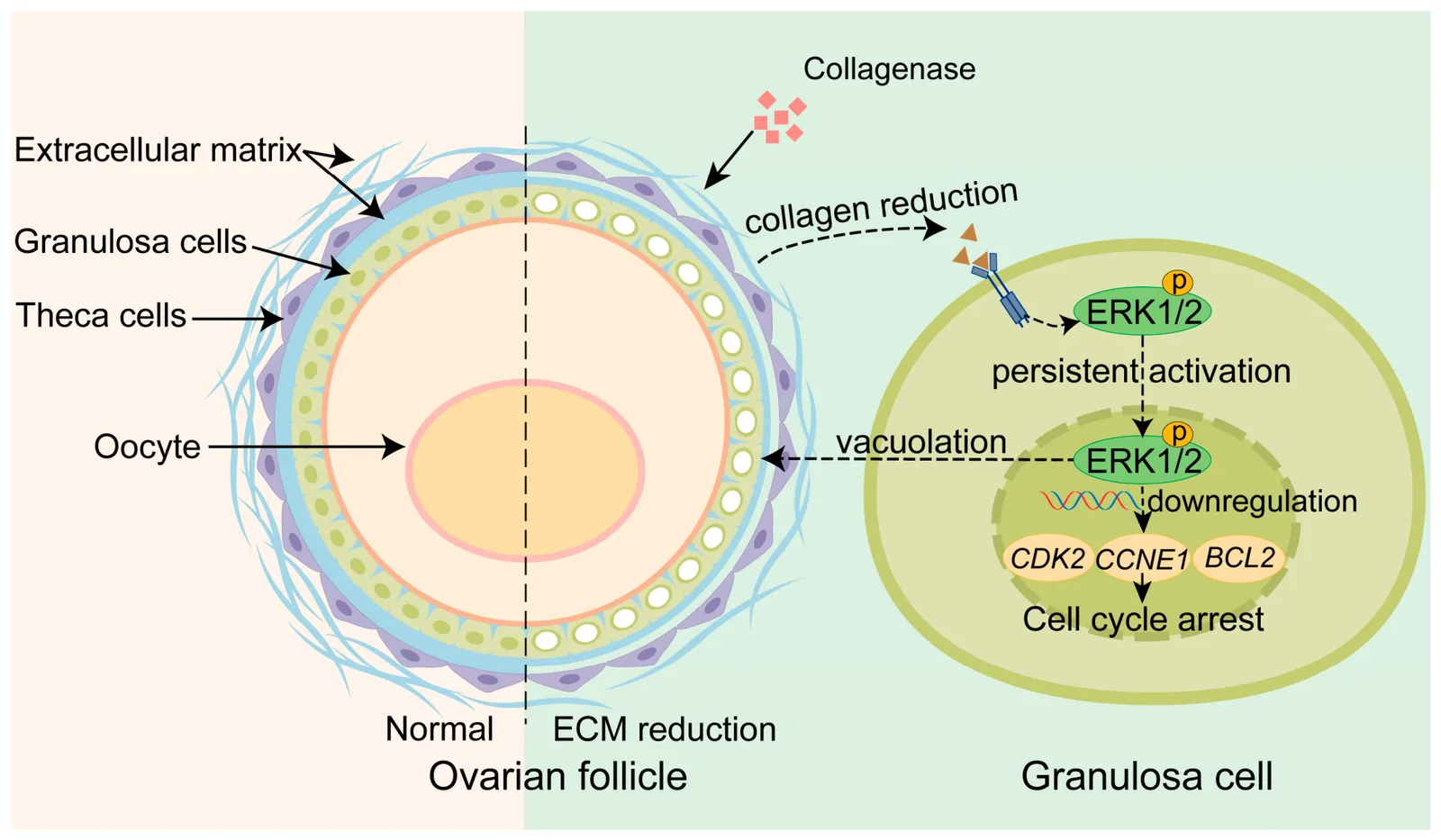
Proposed working model describing the regulatory mechanism by which collagen reduction restricts granulosa cell proliferation. Reduced collagen abundance induces granulosa cell shedding and nuclear vacuolation, accompanied by persistent ERK1/2 activation. This persistent pathway stimulation suppresses the transcription of *CCNE1*, *CDK2*, and *BCL2*, ultimately driving cell cycle arrest and repressing granulosa cell growth. Solid arrows represent validated regulatory relationships, whereas dashed arrows indicate putative molecular associations in this model.

To our knowledge, this is the first study to suggest that collagen regulates granulosa proliferative capacity via the ERK1/2 signaling pathway within an experimentally manipulated in vitro system using ovarian tissues and granulosa cells of *Pelodiscus sinensis*. In vitro collagen reduction treatment using collagenase was found to trigger cell cycle stagnation and repress granulosa cell expansion, a biological phenotype correlated with persistent 24 h ERK1/2 hyperphosphorylation. Based on the present findings, we propose a working model summarizing the collagen–ERK1/2 regulatory cascade observed in our in vitro experiments (Figure 6). This schematic represents only a hypothetical signaling framework rather than a fully validated definitive pathway. These findings, obtained from an in vitro experimental model, may not fully mirror the situation in the intact ovary. Nevertheless, all observations derived from this artificial cell and tissue culture system offer theoretical support for optimizing ex vivo ovarian culture protocols and advancing artificial breeding techniques for chelonian aquatic species.

## Figures and Tables

**Table 1:** Primers for RT-qPCR.

Primer Name	Target Gene	Sequence (5′ to 3′)	GenBank Accession Number
*GAPDH-F*	*GAPDH*	GGCTTTCCGTGTTCCAACTC	NM_001286927
*GAPDH-R*		GACAACCTGGTCCTCCGTGTATC	
*COL1A1-F*	*COL1A1*	GATGTGTGGAAACCGGAACC	XM_075918435.1
*COL1A1-R*		GTCTCCTCTGGGTCCCTCTA	
*COL1A2-F*	*COL1A2*	CCACCAGGAGAAAGGGGTCTA	XM_006114489.4
*COL1A2-R*		CATGGGTCCTTGGCCGTAAT	
*COL4A1-F*	*COL4A1*	CGCTCATGGCTTCCTCGTTA	XM_075918579.1
*COL4A1-R*		CTCCCTGCTGTGCCCAAATC	
*COL4A3-F*	*COL4A3*	GAAAAGGGAGAACGAGGTGC	XM_075937588.1
*COL4A3-R*		ATCATTATGCCCGGCGTACC	
*COL4A5-F*	*COL4A5*	CAAGGTACACAGGGACCCAC	XM_075940947.1
*COL4A5-R*		CAGCTCCCAGCAGTACCTAAG	
*CCNE1-F*	*CCNE1*	AGCTGATGTGGCGACTTTCC	XM_075940176.1
*CCNE1-R*		CCCAGAATTGGTAGCGGTGA	
*CCND2-F*	*CCND2*	AAGTTGCGTTGTGGCTACTG	XM_014573619.3
*CCND2-R*		CCAAAGAGATCTCCCTCTGCAA	
*CDK2-F*	*CDK2*	GCAAGATGCCACCAGATCCT	XM_075901929.1
*CDK2-R*		TGGAACCACAGTGATCGACG	
*BCL2-F*	*BCL2*	TGTGTGGAGAGTGTCCACCG	XM_006122882.4
*BCL2-R*		TCCACAAAGGCATCCCAACC	

## Data Availability

Upon manuscript receipt, the data will be made available upon request.

## Supplementary Materials

The following supporting information can be downloaded at https://www.mdpi.com/article/10.3390/ani16162499/s1, Figure S1: Collagen reduction inhibits granulosa cell proliferation via the ERK1/2 pathway; Figure S2: Original Western blotting images of collagen-related protein expression in follicles of different sizes; Figure S3: Original Western blotting images of COL1A1 and GAPDH protein expression in ovarian tissues following 24 h of collagenase treatment; Figure S4: Original Western blotting images of p-mTOR, mTOR, p-ERK1/2, ERK1/2, and β-actin protein expression in ovarian tissues following 30 min of collagenase treatment; Figure S5: Original Western blotting images of p-mTOR, mTOR, p-ERK1/2, ERK1/2, and GAPDH protein expression in ovarian tissues following 1.5 h of collagenase treatment; Figure S6: Original Western blotting images of p-ERK1/2, ERK1/2, and GAPDH protein expression in ovarian tissues following 1.5 h of co-treatment with collagenase and SCH772984; Figure S7: Original Western blotting images of p-ERK1/2, ERK1/2, and GAPDH protein expression in ovarian tissues following 24 h of co-treatment with collagenase and SCH772984; Figure S8: Original Western blotting images of p-ERK1/2, ERK1/2, and GAPDH protein expression in ovarian tissues following 1.5 h of co-treatment with collagenase and ERK1/2 inhibitor I.
