# Supplementary material for: Ovarian Collagen Regulates Granulosa Cell Proliferation Through Persistent Activation of the ERK1/2 Pathway in the Chinese Soft-Shelled Turtle (Pelodiscus sinensis)
Source: Animals (Basel). 2026 Aug 11;16(16):2499. doi: 10.3390/ani16162499 (PMC13508893; doi:10.3390/ani16162499)
Supplement: Supplementary file 1 [file animals-16-02499-s001.zip › animals-4458844-Supplementary materials.pdf]

Supplementary materials:

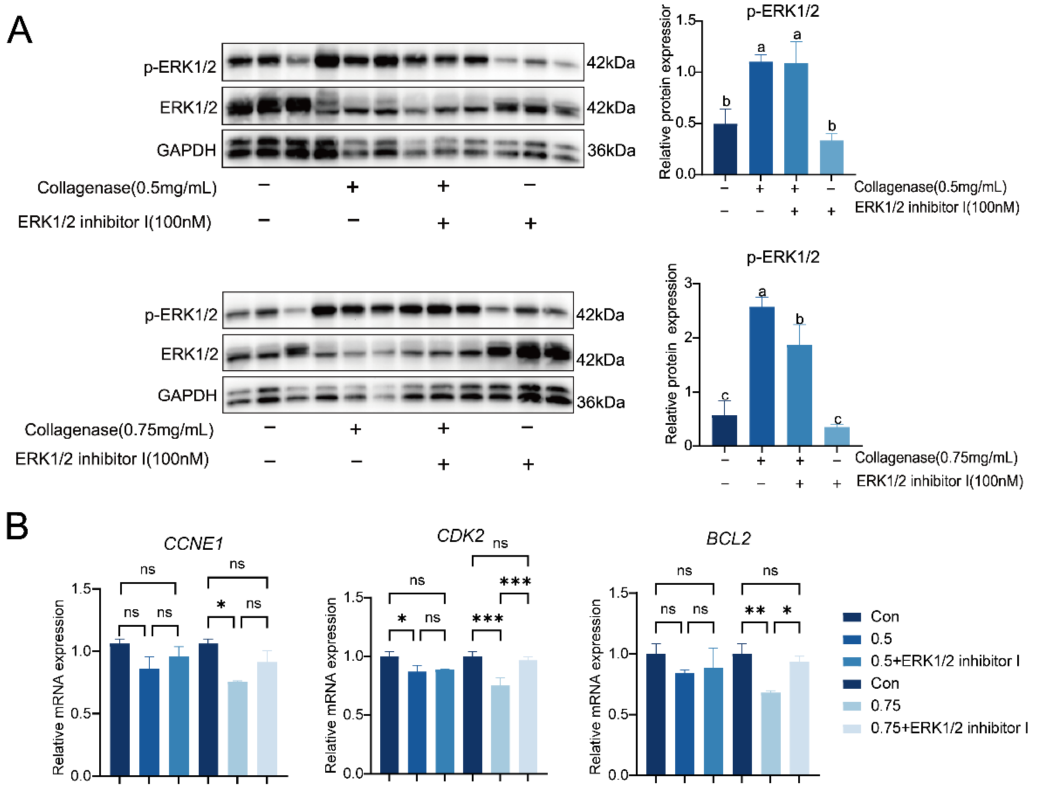

Figure S1 Collagen reduction inhibits granulosa cell proliferation via the ERK1/2 pathway. **(A)** Phosphorylation levels of ERK1/2 in ovarian tissues following 1.5 h co-treatment with collagenase and ERK1/2 inhibitor I. **(B)** Relative mRNA expression of *CCNE1*, *CDK2*, and *BCL2* in ovarian tissues after 24 h co-treatment with collagenase and ERK1/2 inhibitor I. Statistical analysis: one-way ANOVA followed by Tukey's multiple comparison test. $n \geqslant 3$ biological replicates. Statistical significance: Different lowercase letters indicate significant differences between groups ($p < 0.05$); *$p < 0.05$, **$p < 0.01$, ***$p < 0.001$.

Figure 2C-Original Western Blot Images

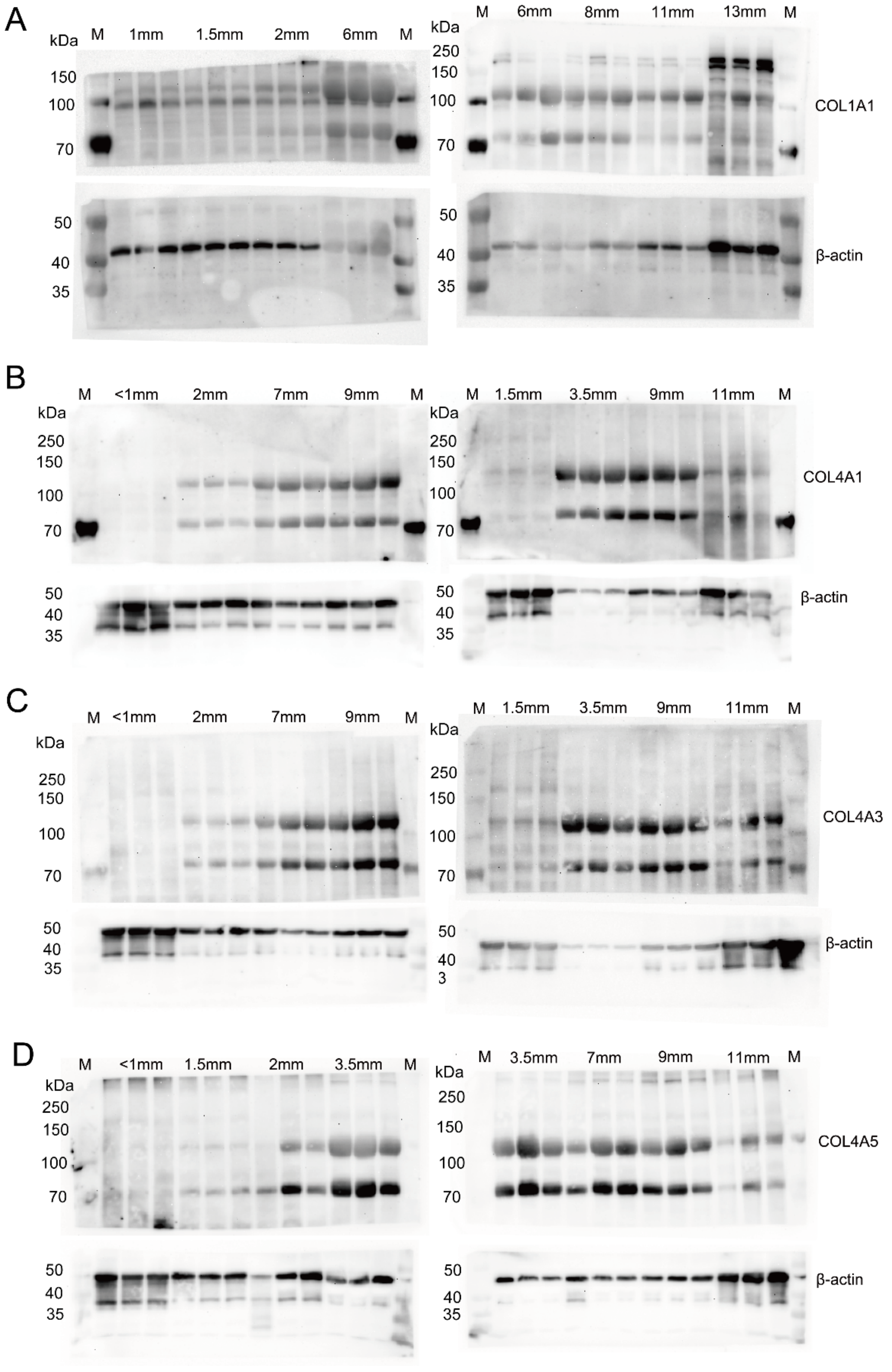

Figure S2 Original Western blotting images of collagen-related protein expression in follicles of different sizes (Figure 2C). **A-D：** COL1A1, COL4A1, COL4A3, and COL4A5 proteins with their corresponding β-actin, respectively. M: Marker.

Figure 4D-Original Western Blot Images

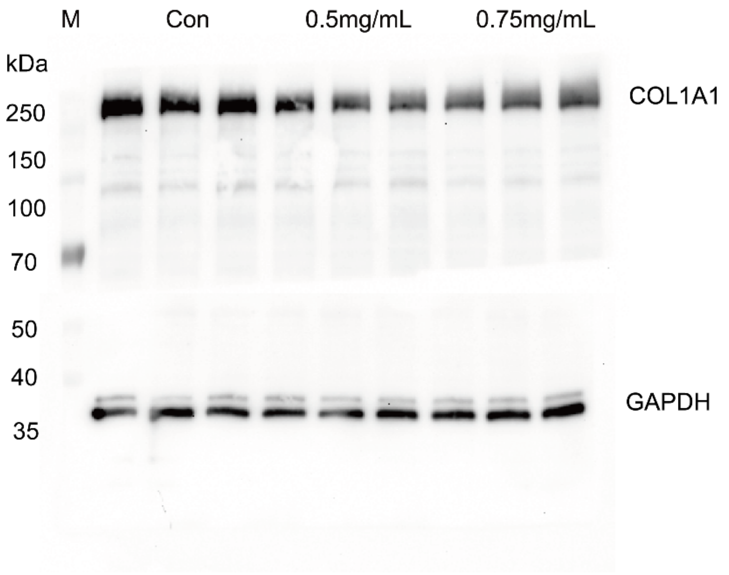

Figure S3 Original Western blotting images of COL1A1 and GAPDH protein expression in ovarian tissues following 24 h of collagenase treatment (Figure 4D). M: Marker.

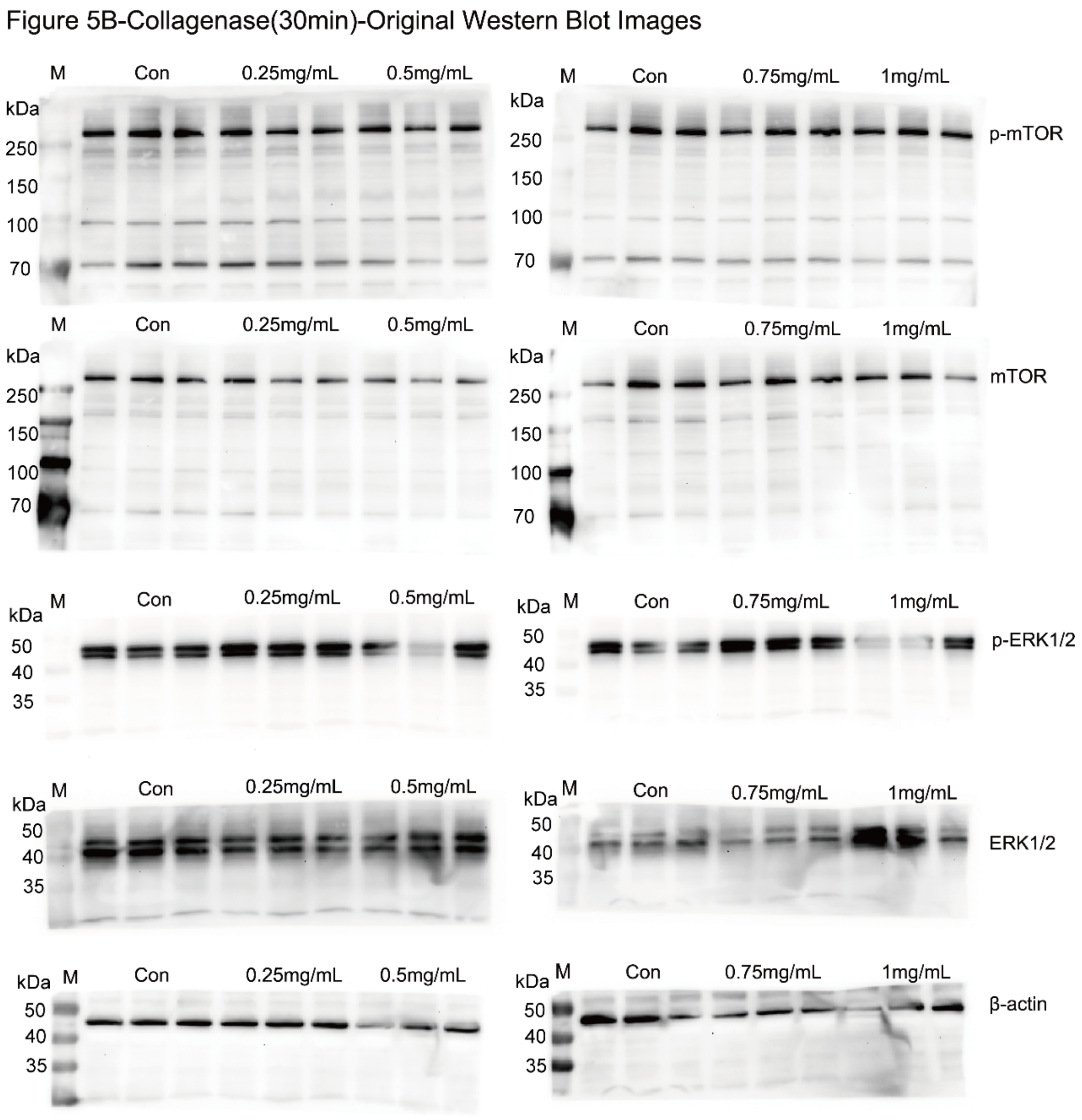

Figure S4 Original Western blotting images of p-mTOR, mTOR, p-ERK1/2, ERK1/2, and β-actin protein expression in ovarian tissues following 30 min of collagenase treatment (Figure 5B). M: Marker.

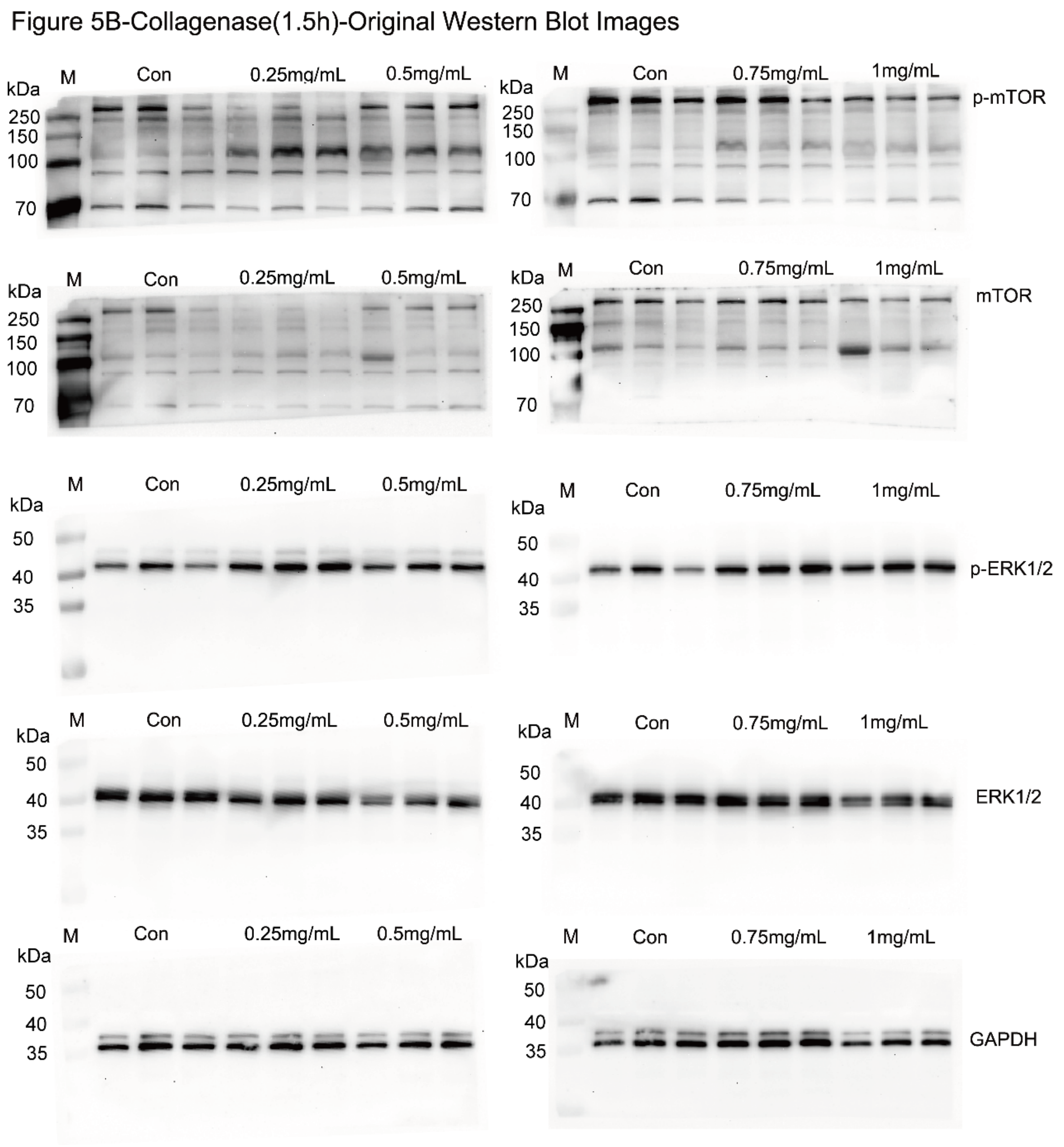

Figure S5 Original Western blotting images of p-mTOR, mTOR, p-ERK1/2, ERK1/2, and GAPDH protein expression in ovarian tissues following 1.5 h of collagenase treatment (Figure 5B). M: Marker.

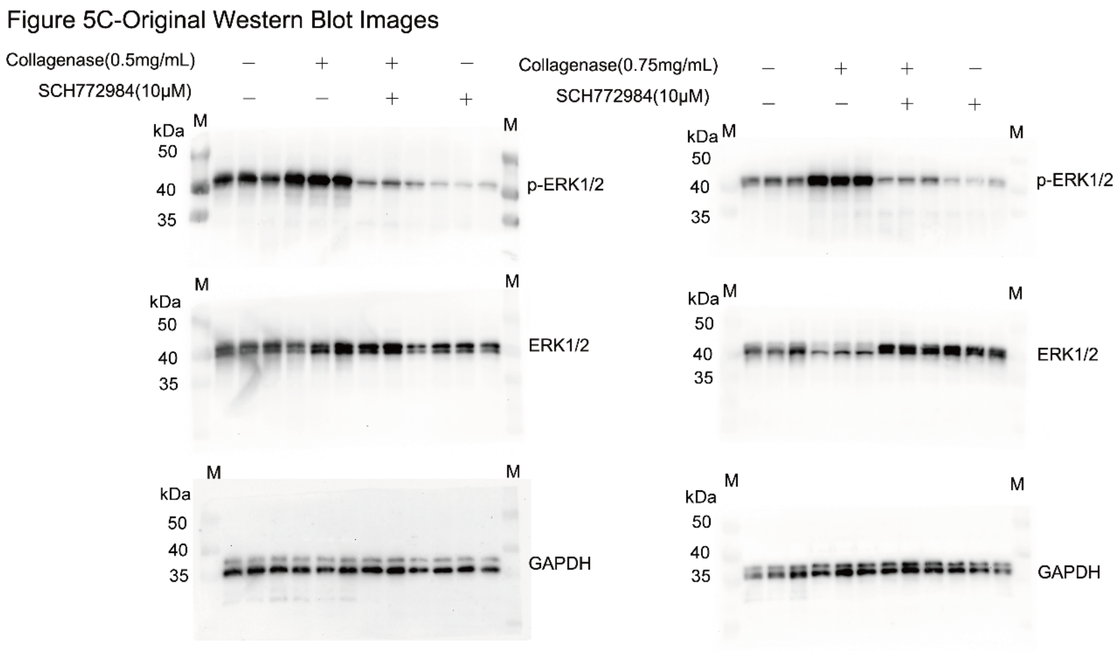

Figure S6 Original Western blotting images of p-ERK1/2, ERK1/2, and GAPDH protein expression in ovarian tissues following 1.5 h of co-treatment with collagenase and SCH772984 (Figure 5C). M: Marker.

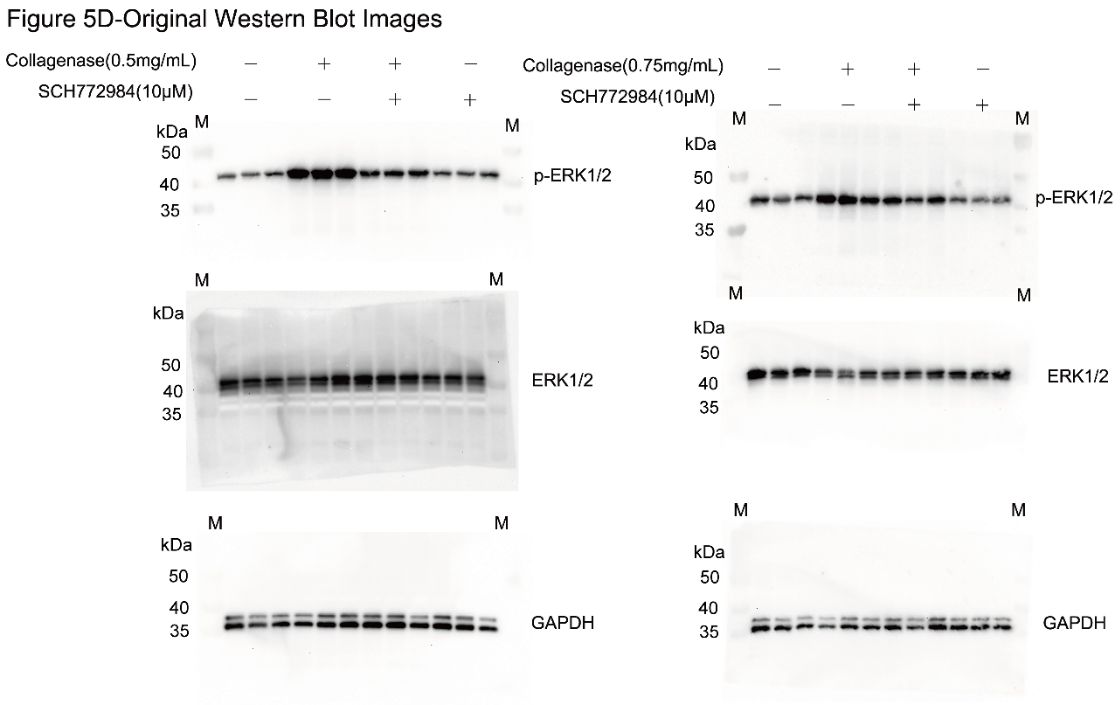

Figure S7 Original Western blotting images of p-ERK1/2, ERK1/2, and GAPDH protein expression in ovarian tissues following 24 h of co-treatment with collagenase and SCH772984 (Figure 5D). M: Marker.

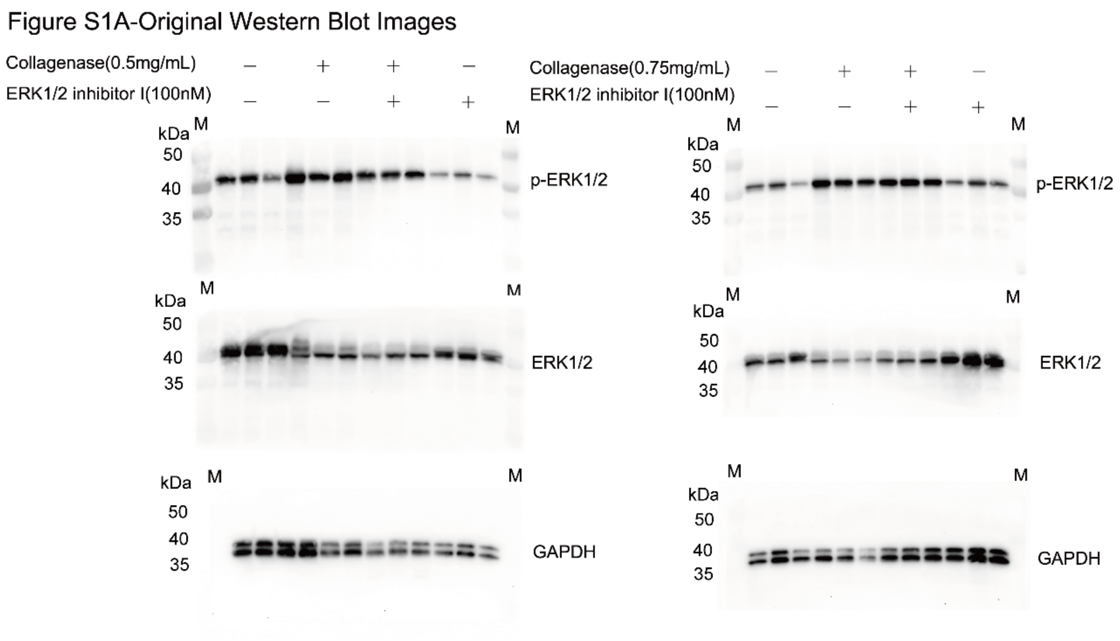

Figure S8 Original Western blotting images of p-ERK1/2, ERK1/2, and GAPDH protein expression in ovarian tissues following 1.5 h of co-treatment with collagenase and ERK1/2 inhibitor I (Figure S1A). M: Marker.
